# SARS-CoV-2 spread in different biosocial strata in Russia in 2020: Groups of risk and victimised groups

**DOI:** 10.7189/jogh.11.03066

**Published:** 2021-04-24

**Authors:** Konstantin S Sharov

**Affiliations:** Koltzov Institute of Developmental Biology of Russian Academy of Sciences, Moscow, Russia

## NOVEL RUSSIAN ANTI-MIGRATION LAW RELATED TO COVID-19 PANDEMIC

Recently several alterations of public policy in migration and asylum provision for refugees, were introduced as parts of a new COVID-19-related Russian federal health care legislation. The main of them are 1) Federal Laws No. 58-FZ of 18 March 2020; 63-FZ of 18 March 2020; 134-FZ of 24 April 2020; 209-FZ of 13 June 2020 “About introducing changes to the Federal Law ‘About the citizenship of Russian Federation;’” 2) Decree of the President of the Russian Federation No. 580 of 23 September 2020 “About introducing changes to the Decree of the President no. 274 of 18 April 2020 ‘About temporary measures of regulating legal situation of foreign citizens and persons without citizenship in the Russian Federation in regard to the threat of further spreading the novel coronavirus infection;’” and 3) ordinance of the Government of the Russian Federation No. 1428 of 15 September 2020 “About implementing measures of safety by a foreign citizen or person without citizenship on the territory of the Russian Federation” [1,2].

These novel legal acts substantially reduce civil rights of working migrants and refugees. New visa restrictions, stricter migration and border control as well as elevated requirements of financial guarantees, have resulted in impaired access of non-Russian citizens, primarily working migrants, to medical care [3]. It is to emphasise that working migrants usually have very limited financial opportunities and almost lack social guarantees in Russia that Russian citizens benefit from [4-7]. The pandemic made the situation even more uncertain for them. Usually the migrants and their relatives, especially from Central Asian region, are being in the permanent state of transboundary movements to and from their native cities and villages [8,9]. This mainly ensures their livelihood and access to medical care that is often available only in their home countries [10,11]. The laws recently introduced undermine the possibilities of migrants/refugees to receive due and timely medical help. This reduction of health protection may create new biosocial risk groups in Russia in the situation of COVID-19 pandemic.

## LEGISLATION- AND MEDIA-DRIVEN HEALTH INEQUITY

In some regions of Russia and within several occupations (eg, in Moscow in the following underpaid professions: taxi drivers; construction site workers; yard and street cleaners; day house cleaners and charladies; grocery market sellers; dockers; loading workmen) the proportion of non-Russian migrants, that predominantly arrive from Central Asia, Middle East, Ukraine, and Moldova, may amount to seventy-eighty per cent of the workforce [4]. Hence, the problem of COVID-related health care equality needs to be addressed urgent.

We trust that many of the new legal initiatives may be emotionally-dictated rather than evidence-based and their adoption mainly accounted for by a common belief that migrants represent one of the riskiest epidemiological groups during the COVID-19 pandemic. This belief is widely represented on TV channels, including “official,” government-sponsored channels, and in social networks, both in Russia [5] and Europe [12,13]. Media, including social networks, may significantly amplify the misbelief about the elevated epidemiological risks associated with migrants and refugees, their life style and working habits [14-17]. It is not uncommon to find praises of the new restrictive laws in social networks, whereas increasing epidemiological risks largely proceeds from the laws in question.

**Figure 1:**
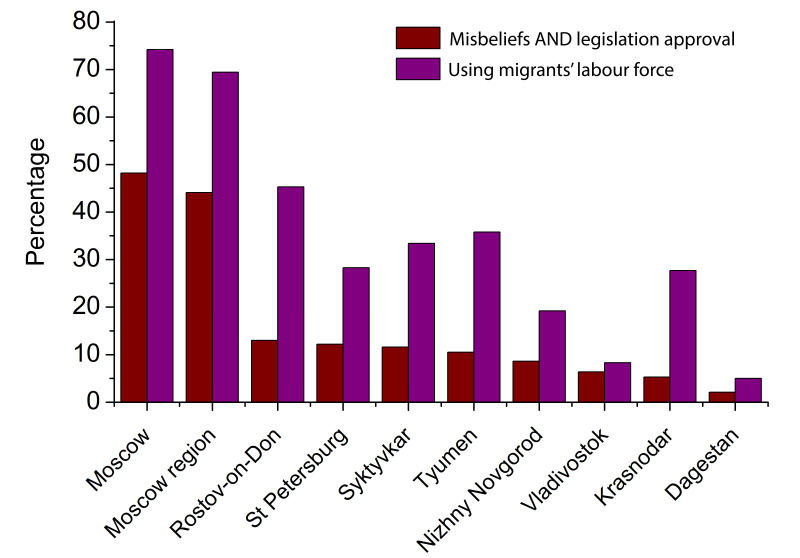
Comparison of the level of COVID-related epidemiological misbeliefs towards non-Russian (by their ethnic origin or citizenship) working migrants and refugees in different regions of Russia (with simultaneous social support of anti-migrant- and anti-refugee legal initiatives) (brown bars) with the level of using cheap migrant labour force (purple bars). Percentage estimations demonstrated as brown bars are based on population rate of different regions of Russia, inclusion of different regions in Internet community, and the results of our social media analysis (146 204 accounts in social networks). Migrant labour force is taken into consideration in low-qualification areas (taxi drivers, construction site workers, yard and street cleaners, day cleaners and charladies, grocery market sellers, dockers, loading workmen). The levels of using migrant labour force is taken from the source [4]. Pearson correlation coefficient between values in brown and purple columns *C* is 0.9219 at *P* less than or equal to 0.000148 (very strong correlation) and Kendall correlation coefficient *C*_Kend_ is 0.7778 at *P* less than or equal to 0.00175.

In October 2020 – March 2021, we performed a content analysis of 1 271 052 open personal accounts of Russian segments of social networks VK, Facebook, Instagram, Odnoklassniki (“School Mates”) and Twitter in regard to misbeliefs about non-Russian working migrants and refugees being the main spreaders of SARS-CoV-2 in Russia. The choice of accounts was randomised. 235 405 accounts (18.5%) contained signals of the account holder’s misbeliefs about non-Russian migrants and refugees being super-spreaders of SARS-CoV-2. Of this set, in 146 204 cases (62.1%) we detected direct social support of anti-migrant and anti-refugee novel legal initiatives related to the COVID-19 pandemic. The content analysis was carried out with the help of proprietary C++-based programme that performed social network screening. The results are presented in **Figure 1****.** One may see strong correlation between the level of social support of the new anti-migrant restrictive legislation and the level of using migrants’ labour force in a region. It is likely that negative emotions towards migrants and refugees are mainly caused by social tensions, work competition and psychological antipathy of a considerable part of general population in the regions with the highest level of using migrants’ labour force, not by rational assessment of epidemiological information and expectations. The main most frequent misbeliefs detected during the content analysis of social media are summarised in **Table 1**. The adoption of the new restrictive laws may be partially explained by these epidemiological fears.

**Table 1:** Ten most frequent misbeliefs about the epidemiological hazards of the “invaders,” migrants and refugees of non-Russian ethnic origin or non-Russian nationality (results of analysing the set containing 235 405 accounts in social networks)

No	Misbelief	Mean frequency in the social network accounts analysed
**1**	They (henceforth migrants and refugees) are dirty by their nature and origin and, therefore, they are super-spreaders of the new coronavirus	64.2%
**2**	They live in overpopulated conditions in slum districts and suburban areas near large cities, that may be new coronavirus niduses. These settlements and camps must be destroyed and disinfected while their inhabitants must be cast off	55.8%
**3**	Before arriving to the new “promised land,” they lived in highly contagious regions of Central Asia, Africa and Near/Middle East, that may contain multiple very dangerous strains of the novel coronavirus SARS-CoV-2	54.7%
**4**	Their children are infectious disease-stable and this immunity has been developing in their “wild” countries for centuries. On interacting with our children in schools and public places, their children transmit the novel coronavirus, but do not fall ill themselves	48.8%
**5**	They must be compelled to leave new places of residence and return to their native countries by strict administrative decrees based on the “evident” epidemiological information	41.2%
**6**	They must not be provided with the medical help available within public medical insurance programmes. It will not help them, but it creates additional pressure on the Russian state budget	32.5%
**7**	Now we experience shutdown of economies and get poorer every day. Their social allowances must be revoked in our favour till the end of the COVID-19 pandemic	28.4%
**8**	They will deprive us of anti-SARS-CoV-2 vaccines and antiviral medicines that we would otherwise have ourselves	26.3%
**9**	Migration routes from Asia were the main sources of entering the coronavirus into Russia. The countries that prohibited admittance of migrants to their territory, are much less stricken by COVID-19	25.9%
**10**	They must be placed in new ghettoes with their own schools, canteens and lavatories. Admitting them in public places will not ever give us the opportunity to stop the coronavirus	17.6%

## DATA ON ANTI-SARS-COV-2 IMMUNOGLOBULIN TESTING WITHIN DIFFERENT BIOSOCIAL STRATA

The data on SARS-CoV-2 population infection rate within various biosocial strata in Russia, based on our analysis of 45 652 anti-SARS-CoV-2 *IgG* test results made in May-December 2020, may offer a different view. Test results of refugee camps in Southern Russia were included (Rostov-on-Don region for refugees from Ukraine and Dagestan for refugees from the recent Armenia-Azerbaijani war in Nagorny Karabakh).

Demographic data collected included nationality, ethnic origin, race, gender and age. The age range was 12-88 years for the whole set.

**Figure 2:**
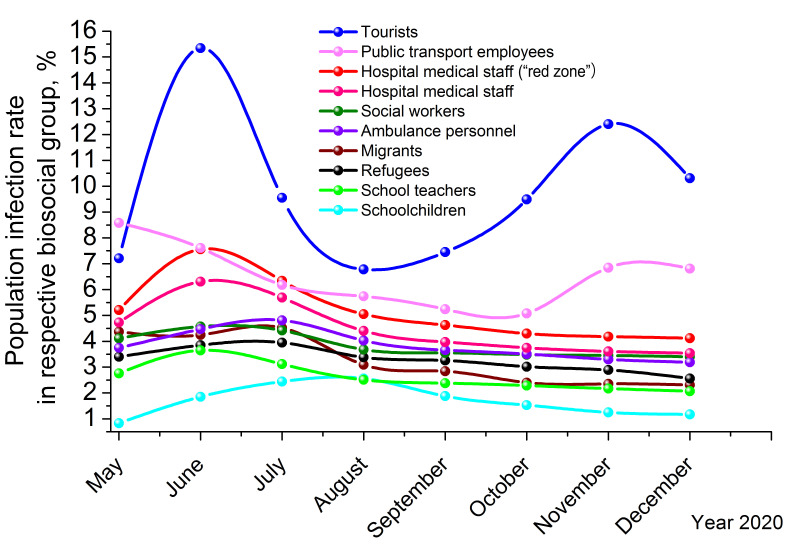
Population infection rate (*PIR*) dynamics for different biosocial strata, since May to December 2020.

We investigated the following risk groups: tourists, public transport employees, hospital medical staff, hospital medical staff (“red zone”), social workers, ambulance personnel, working migrants, refugees seeking political asylum, school teachers and schoolchildren. They were chosen on the basis of the works [18-21]. These works assessed the level of COVID-19 risk in different biosocial groups in terms of transmission by asymptomatic and paucisymptomatic carriers as well as by alleged “super-spreaders.” The authors supposed that super-spreading SARS-CoV-2 mainly took place 1) in communities with traditional life style; 2) in the “red zone” of COVID-19 infirmaries; 3) during transboundary relocation of people; 4) by aged persons; and 5) by children. However, in the works cited there was no comparison of these groups with each other. In our study, we determined in-group population infection rate (*PIR*, %) in the risk groups on the basis of epidemiological data of antibody testing. It may be an appropriate instrument of SARS-CoV-2 transmission comparison in different biosocial groups. In **Figure 2**, *PIR* is presented for the groups studied.

**Figure 2** demonstrates that the curves have parts of growth and decay. Decays may be explained by the increase in testing set size and, therefore, the results becoming more correct and representative with time. Ideally, all decays should have an inverted exponential character: *PIR ~ a + be^–t^*, where *t* is time and *a,b* are numerical coefficients of proportionality. Growths in the graph may represent an increase in the spread of SARS-CoV-2 infection within respective biosocial groups and/or the introduction of more antibody testing.

In **Figure 2**, two COVID-19 pandemic waves may be detected, the first in May-July 2020 and the second in October-December 2020. The second wave led to the serious surge of in-group *PIR* for the strata of tourists and public transport employees (including airport personnel), whereas for other strata, including working migrants and refugees, such tendency was not observed. The major conclusion deduced from **Figure 2**, is that migrants and refugees do not contribute to spreading SARS-CoV-2 in Russia significantly. Their in-group *PIR* dynamics is between school teachers and ambulance medical personnel, and mainly coincides with *PIR* dynamics calculated for the stratum of social workers. Point-by-point analysis of the data in the antibody testing set collected in ten Russian regions studied (Moscow, Moscow region, St Petersburg, Nizhny Novgorod, Krasnodar, Dagestan, Syktyvkar, Rostov-on-Don, Tyumen, Vladivostok), shows that there is almost no correlation between the refugee/migrant status of a person and probability of his/her being infected with SARS-CoV-2 (average Spearman correlation coefficient *C* = 0.2716 at *P* less than 0.4431).

## CONCLUSION

The main contribution to spreading SARS-CoV-2 in Russian population is made by tourists (upper blue line in **Figure 2**). Therefore, we may conclude that tourists are one of the most risky biosocial strata in COVID-19 epidemiological aspect and hence tourist relocation should be thoroughly controlled. This notwithstanding, working migrants and refugees are mainly victimised in the novel federal migration legislation.

**Figure:**
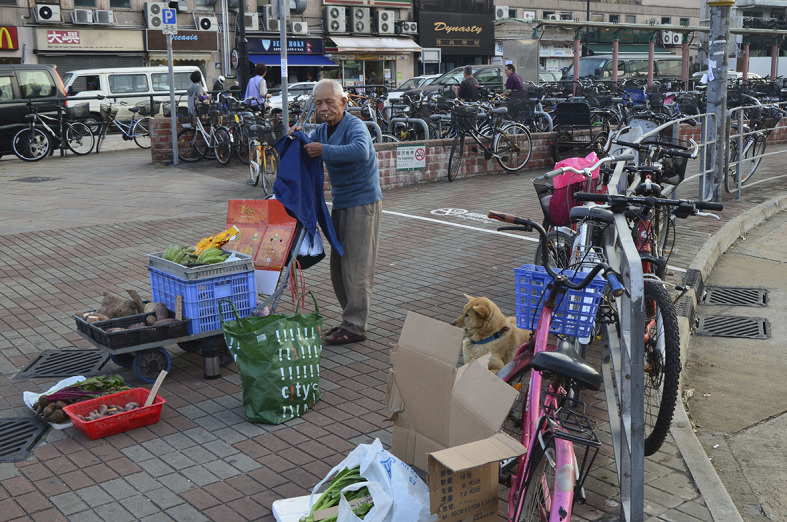
Photo: A trading quarter of Chinese migrants to the Russian Far East (from the author’s own collection, used with permission). In this region, the majority of working migrants is comprised by Chinese. Due to their rather traditional life style and frequent engagement in food trade/delivery/cooking/catering, especially in the cities of Vladivostok, Khabarovsk, Blagoveshchensk and Komsomolsk-upon-Amur, they were often labelled as “super-spreaders” of SARS-CoV-2 in 2020 by Russian press, social network users and sometimes even administrative bureaucrats. The parallel was regularly drawn with the food market in Wuhan where the initial outbreak of the novel coronavirus was registered in 2019.

We suggest that the new Russian COVID-19-related migration legal amendments should be re-considered to support health equity in Russia. These laws create the situation of serious health inequity to non-Russian citizens, mainly working migrants and political refugees, and, from the author’s viewpoint, should be revoked. Introduced as legal instruments to limit the SARS-CoV-2 spread, they may instead contribute to its more dramatic impact on Russian society.

What can be done in such a situation to attract the legislators’ attention? The activities of health care-related non-commercial, social and non-governmental organisations may be crucial, they could engage in fundraising and providing basic medical help. Constructing inclusive narratives in the media, including social networks, is also a promising choice, which would counteract the common misbelief about the epidemiological “danger” of migrants and refugees.

Several publicly funded social initiatives that would promote health equality to the migrants and refugees during the pandemic, were recently launched in the Russian Centre for Racial and Ethnic Equality created as an interuniversity venue. Shooting documentary films about refugees, recording musical performances, holding art exhibitions, making short video reports for fundraising and placing the complete relevant epidemiological information in Internet websites easily accessible from social networks, are the main goals. The Centre works in collaboration with Teranga project launched and supervised in Italy by Sophia Seymour, Daisy Squires and Lou Marillier [22].

Similar projects may be effectively used to draw the attention of general population, media and policy makers to the unsolved problem of the COVID-19 pandemic-caused victimisation by new migration regulations that place millions of people without Russian citizenship in the situation where they cannot afford to apply for and receive necessary medical help.
